# Clinical Relevance and Drug Modulation of PPAR Signaling Pathway in Triple-Negative Breast Cancer: A Comprehensive Analysis

**DOI:** 10.1155/ppar/4164906

**Published:** 2024-12-21

**Authors:** Yanxia Zhang, Yunduo Liu, Mei Zhang, Guanjie Li, Siling Zhu, Keping Xie, Bin Xiao, Linhai Li

**Affiliations:** ^1^Department of Laboratory Medicine, The Sixth School of Clinical Medicine, The Affiliated Qingyuan Hospital (Qingyuan People's Hospital), Guangzhou Medical University, Qingyuan, China; ^2^School of Medicine, The South China University of Technology, Guangzhou, China; ^3^School of Public Health, Dali University, Dali, China; ^4^Thyroid and Breast Specialty of General Surgery Area Five, The Sixth School of Clinical Medicine, The Affiliated Qingyuan Hospital (Qingyuan People's Hospital), Guangzhou Medical University, Qingyuan, China; ^5^Department of Laboratory Medicine, Guangdong Provincial Second Hospital of Traditional Chinese Medicine (Guangdong Provincial Engineering Technology Research Institute of Traditional Chinese Medicine), Guangzhou, China; ^6^The Fifth College of Clinical Medicine, Guangzhou University of Chinese Medicine, Guangzhou, China

## Abstract

Triple-negative breast cancer (TNBC) is highly heterogeneous and poses a significant medical challenge due to limited treatment options and poor outcomes. Peroxisome proliferator-activated receptors (PPARs) play a crucial role in regulating metabolism and cell fate. While the association between PPAR signal and human cancers has been a topic of concern, its specific relationship with TNBC remains unclear. Integrated analysis of large published datasets from clinical cohorts and cell lines through databases has proven to be a powerful and essential approach for understanding cancer and uncovering new molecular targets. Here, we conducted a comprehensive study investigating the clinical relevance and drug modulation of the PPAR signaling pathway in TNBC, using data from The Cancer Genome Atlas (TCGA) for TNBC patients and Genomics of Drug Sensitivity in Cancer (GDSC) for TNBC cell lines, along with drug perturbation information from Connectivity Map (CMap). In the TCGA-TNBC cohort, higher PPAR signaling activity was not associated with clinical stage, prognosis, tumor mutational burden, microsatellite instability, homologous recombination deficiency, stemness, or proliferation status. However, it was linked to older age; an elevated rate of piccolo presynaptic cytomatrix protein (PCLO) mutations; and oncogenic signal transduction involving MAPK, Ras, and PI3K-Akt pathways. Additionally, it influenced biological pathways including fatty acid metabolism, AMPK signaling, and ferroptosis. Strikingly, higher PPAR activity appeared to promote the formation of an antitumor immune and microbial microenvironment. In the GDSC-TNBC cells, nevertheless, it seemed to incur chemoresistance. Furthermore, we identified a batch of potential compounds that can regulate the PPAR signaling pathway. Lastly, our experimental validation demonstrated the ability of the histone deacetylase (HDAC) inhibitor chidamide to activate the PPAR signal in TNBC cells. In conclusion, the PPAR signaling pathway likely has pleiotropic biological effects in TNBC. These preliminary but interesting findings enhance our understanding of the role played by PPAR signal and provide new insights into the heterogeneity driven by it in TNBC.

## 1. Introduction

Breast cancer stands as the most prevalent cancer and the leading cause of cancer-related mortality among women globally [1]. Triple-negative breast cancer (TNBC), identified by the absence of estrogen receptor, progesterone receptor, and HER2 expression, represents 15%–20% of all breast cancer cases [2]. Its aggressive nature and limited treatment options often result in poor prognoses [3]. The high heterogeneity of TNBC presents a significant hurdle for tailored therapeutic approaches [4]. Efforts have focused on classifying TNBC into molecular subtypes with distinct mutational profiles, genetic alterations, and biological processes to guide clinical decision-making and advance precision medicine [5–9].

Peroxisome proliferator-activated receptors (PPARs), discovered in 1990, belong to the nuclear hormone receptor superfamily of transcription factors [10] and are significant regulators of metabolism and cell fate [11]. While attention has been paid to the potential connections between PPARs and human cancers [12, 13], their roles in cancer development remain complex and context dependent [14–16]. Limited information is available regarding the role of PPAR signal in TNBC. Recent studies have identified the involvement of PPAR signal in metabolic reprogramming [17] and in driving the death of TNBC cells under specific conditions [18]. Further research is needed to deepen our understanding of the role played by PPAR signal in TNBC. Given the heterogeneity of TNBC in pathology, genomic alteration, gene expression, and the tumor microenvironment (TME), we sought to investigate whether the degree of PPAR signaling pathway activity might be associated with either oncogenic or tumor suppressive properties. Moreover, emerging evidence highlights the significant roles of intratumor microbiota in cancer pathogenesis [19]. Metabolic regulation stands out as an important mechanism mediating the interaction between the host and the microbe [20]. A recent study has further unveiled the correlation between breast intratumor microbiota and the heterogeneity of host metabolism—a significant mechanism regulated by PPARs in humans [21]. Therefore, it is essential to explore the potential association between PPAR signaling pathway activity and microbiota in human breast cancer in this study.

With the advancements in high-throughput sequencing technology and bioinformatics, cancer-related genomic and transcriptomic data have been integrated on an unprecedented scale in public databases. These databases have proven to be valuable resources for breast cancer research, facilitating tasks such as subtype definition, prognostic signature revelation, and identification of molecular or signaling targets [22]. We systematically examined the relationship between PPAR signaling pathway activity and multiple clinical or genetic characteristics in samples from TNBC patients obtained from The Cancer Genome Atlas (TCGA) [23]. Additionally, we leveraged data from Genomics of Drug Sensitivity in Cancer (GDSC) [24] to understand TNBC's response to therapy and explored drug sensitivity alterations involving the PPAR signaling pathway. What is more, our analysis identified several drug candidates capable of activating or inhibiting PPAR signal. Lastly, cellular experiments confirmed the potential of the histone deacetylase (HDAC) inhibitor chidamide to activate PPAR signal in TNBC cells. This investigation may offer valuable insights into the clinical relevance and drug modulation of the PPAR signaling pathway, with significant potential to inform clinical decision-making in the treatment of individual TNBC patients.

## 2. Materials and Methods

### 2.1. Acquisition of Publicly Available Data for TNBC Patients and Cell Lines

Transcriptomic data, somatic mutation profiles, and clinical information for TNBC patients were obtained from TCGA (https://www.cancer.gov/tcga/) portal. Additionally, microbiomic data were downloaded from the cBioPortal (https://www.cbioportal.org/) [25]. Tumor mutational burden (TMB) scores were derived from the built-in dataset of the “maftools” R package [26]. Other corresponding data, including microsatellite instability (MSI) scores [27], homologous recombination deficiency (HRD) scores [28], and stemness indices (mDNAsi and mRNAsi) [29] were obtained from indicated studies. Transcriptomic data and drug sensitivity measurements (half-maximal inhibitory concentration (IC50) values) for TNBC cell lines were sourced from the GDSC (https://www.cancerrxgene.org/) portal.

### 2.2. Acquisition of Reference Gene Sets and Lists

Classical gene sets, including the PPAR signaling pathway from Kyoto Encyclopedia of Genes and Genomes (KEGG) [30], were extracted using the “KEGGREST” R package. Immune-related gene sets were obtained from the ImmPort (https://www.immport.org/) portal [31]. The lists of oncogenes and immune-related and chemoresistance-related genes were sourced from the ONGene (http://ongene.bioinfo-minzhao.org/) portal [32] and published studies [33, 34], respectively.

### 2.3. Pathway Enrichment Analyses

Overrepresentation analysis (ORA) and gene set enrichment analysis (GSEA) [35] were performed using the “clusterProfiler” R package [36] to identify significantly enriched pathways. Gene set variation analysis (GSVA) was conducted using the “GSVA” R package [37] to assess the overall activity of specified pathway.

### 2.4. Survival Analysis

In order to obtain more accurate results, individuals with less than 1 month of overall survival were excluded. The “surv_cutpoint” function from the “survminer” R package determined the optimal cutpoint, stratifying patients into the high and low groups. The Kaplan–Meier curve and log-rank test were performed using the “survminer” and “survival” R packages.

### 2.5. TME Analysis

The StromalScore, ImmuneScore, and ESTIMATEScore of the TME were calculated by the “estimate” R package [38]. Tumor purity was inferred based on the ESTIMATEScore. The infiltration levels of immune cells were assessed using Tumor Immune Estimation Resource (TIMER, http://timer.cistrome.org/) [39] and Cell-type Identification By Estimating Relative Subsets Of RNA Transcripts (CIBERSORT) [40] algorithms. Shannon indices of T cell receptor (TCR) clonotype diversity were sourced from a published study [33]. Immune cytolytic activity (CYT) was determined as the geometric mean of GZMA and PRF1 expression [41].

### 2.6. Microbial Alpha Diversity Estimate

Microbial alpha diversity was estimated using the “vegan” R package. Specifically, four different alpha diversity indices (Chao1, ACE, Shannon, and Simpson) were calculated to analyze and understand the distribution of biodiversity within the microbiomic data.

### 2.7. Connectivity Map (CMap) Analysis

CMap (https://clue.io/) is a tool that allows us to predict the effectiveness of drugs based on gene expression changes [42]. The tool works by assigning a connectivity score (tau) to each drug. A score of 100 means that the drug produces the same effect as the change in the input genes, while −100 means the drug produces an effect opposite to the input genes' change. In the current study, we used all genes in the PPAR signaling pathway as input for a CMap analysis. We considered a tau greater than 90 or lesser than −90 to be significant.

### 2.8. Cell Culture

The human TNBC cell line MDA-MB-231 was obtained from the Shanghai Cell Bank of the Chinese Academy of Sciences. The cells were cultured in DMEM high-glucose medium (Gibco, C11995500CP), which contains L-glutamine, 4.5 g/L D-glucose, 110 mg/L sodium pyruvate, 10% fetal bovine serum (ExCell Bio, FSP500), and 1% penicillin/streptomycin (Gibco, 15140-122). The cultures were maintained at 37°C with 5% CO_2_ in a humidified atmosphere. To prepare for RNA sequencing (RNA-seq), quantitative reverse transcription polymerase chain reaction (qRT-PCR), and Western blot analysis, the cells were exposed to either dimethyl sulfoxide (DMSO; Sigma-Aldrich, D2650) or chidamide (Selleck, S8567) for 24 h.

### 2.9. RNA Library Preparation and Sequencing

We isolated total RNA using TRIzol reagents (Thermo Fisher, 15596018) and then performed messenger RNA (mRNA) extraction using poly-T oligo-attached magnetic beads, followed by fragmentation with divalent cations at an elevated temperature. For first-strand cDNA synthesis, we utilized random hexamer primers and RNase H-M-MuLV Reverse Transcriptase. Subsequently, second-strand cDNA synthesis was carried out using DNA Polymerase I and RNase H. The remaining overhangs were converted into blunt ends using exonuclease and polymerase, and the 3⁣′ ends of DNA fragments were adenylated, followed by ligation of adaptors. Purification of the library fragments, with a preference for a length of 250–300 bp, was accomplished using the AMPure XP system (Beckman Coulter, Beverly, United States). The initial library was then amplified via PCR using Phusion High-Fidelity DNA Polymerase and universal or index primers. The resulting products were purified using the AMPure XP system and assessed on the Agilent Bioanalyzer 5400 system. The cBot Cluster Generation System and Illumina TruSeq PE Cluster Kit v3-cBot-HS were used to cluster the samples. Subsequently, the libraries were sequenced on the Illumina NovaSeq 6000 platform, generating paired-end reads of 150 bp.

### 2.10. Gene Expression Quantification and Differential Analysis

To generate clean reads, we preprocessed our raw data in FASTQ format using fastp v0.23.1, which includes filtering out reads with low quality, poly-N sequences, or adapters. Following that, we mapped the clean reads to the GRCh38 reference genome with HISAT2 v2.0.5 and quantified the reads mapped to each gene using featureCounts v1.5.0-p3. Gene expression levels were assessed using fragments per kilobase million (FPKM), factoring in both gene length and read count. We carried out differential expression analysis using the R package “DESeq2” v1.29.0, adjusting *p* values to maintain control over the false discovery rate (FDR) using the Benjamini and Hochberg method. We considered genes with an FDR < 0.05 and an absolute fold change (FC) > 1.5 to be differentially expressed.

### 2.11. Real-Time Quantitative PCR (RT-qPCR) Analysis

We started by isolating total RNA with TRIzol reagents and then performed reverse transcription into cDNA using Evo M-MLV RT Premix for QPCR (Accurate Biology, AG11706). The subsequent step involved conducting RT-qPCR with the SYBR Green Pro Taq HS qPCR Kit (Accurate Biology, AG11701). For quantification, we employed the 2^(−ΔΔCT)^ method, with *β*-actin serving as the internal reference. The following are the primers used in the procedure: ACOX2 forward: 5⁣′-AGCACCCCGACATAGAGAGC-3⁣′, ACOX2 reverse: 5⁣′-CTGCGGAGTGCAGTGTTCT-3⁣′; ANGPTL4 forward: 5⁣′-CCTCTCCGTACCCTTCTCCA-3⁣′, ANGPTL4 reverse: 5⁣′-AAACCACCAGCCTCCAGAGA-3⁣′; CPT1A forward: 5⁣′-GATCCTGGACAATACCTCGGAG-3⁣′, CPT1A reverse: 5⁣′-CTCCACAGCATCAAGAGACTGC-3⁣′; FABP4 forward: 5⁣′-AGCACCCTCCTGAAAACTG-3⁣′, FABP4 reverse: 5⁣′-GCAAAGCCCACTCCTACTT-3⁣′; FABP6 forward: 5⁣′-ACCGGCAAGTTCGAGATGG-3⁣′, FABP6 reverse: 5⁣′-CCTTTTCGATTACATCGCTGGA-3⁣′; PLIN5 forward: 5⁣′-TGCTGCTCAGCCTGCCATAC-3⁣′, PLIN5 reverse: 5⁣′-AGGACCTTTATTCTGGAGGCAAATC-3⁣′; PLTP forward: 5⁣′-TGATTGACTCCCCATTGAAGC-3⁣′, PLTP reverse: 5⁣′-CGTCCATAGTCATGCTGGACA-3⁣′; PPARA forward: 5⁣′-TTCGCAATCCATCGGCGAG-3⁣′, PPARA reverse: 5⁣′-CCACAGGATAAGTCACCGAGG-3⁣′; SLC27A2 forward: 5⁣′-GTGGAGAAAGATGAACCTGTCCG-3⁣′, SLC27A2 reverse: 5⁣′-CTGAGCCTTTGCTCCAGCATAG-3⁣′; SORBS1 forward: 5⁣′-ATTCCCAAGCCTTTCCATCAG-3⁣′, SORBS1 reverse: 5⁣′-TTTTGCTGTTCTCGATTGTGTTG-3⁣′; *β*-actin forward: 5⁣′-TCACCCACACTGTGCCCATCTACGA-3⁣′, *β*-actin reverse: 5⁣′-TGAGGTAGTCAGTCAGGTCCCG-3⁣′.

### 2.12. Western Blot Analysis

After rinsing the cells, we used lysis buffer to lyse them and measured the protein concentrations using the BCA Protein Assay kit (Beyotime, P0011). Equal amounts of the cell lysate were loaded onto SDS-PAGE and transferred to a PVDF membrane (Millipore). Subsequently, the membranes were incubated overnight at 4°C with primary antibodies and then incubated with appropriate dilutions of secondary antibodies conjugated with horseradish peroxidase (Abcam ab205718 or Cell Signal Technology #7076). We used chemiluminescence detection reagent (Millipore, WBKLS0100) to visualize the immunoreactive bands. Listed are the primary antibodies used: PPAR*α* (Abcam, ab227074, 1:1000), CPT1*α* (Abcam, ab234111, 1:1000), and *β*-actin (Absin, abs132001, 1:5000).

### 2.13. Statistical Analysis

To examine the differences and correlations between groups, we utilized Wilcoxon rank-sum (or Student's *t*) and Pearson's correlation tests via the “wilcox.test” (or “t.test”) and “cor.test” functions in R software, respectively. Statistical significance was set at *p* < 0.05 unless otherwise stated.

## 3. Results

### 3.1. Genetic Landscapes and Clinical Significance of Altered PPAR Signaling Pathway in TNBC

**Figure 1:**
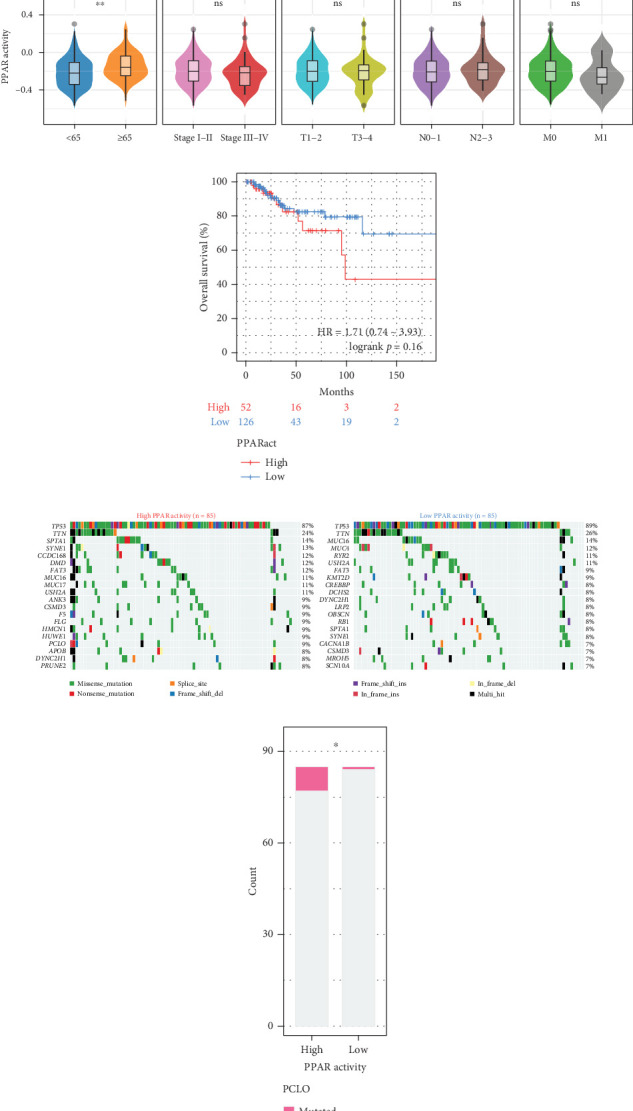
Genetic landscapes and clinical significance of altered PPAR signaling in TCGA-TNBC cohort. (a) Expression pattern of genes in the PPAR signaling pathway and overall PPAR activity (GSVA score) in 190 primary tumors and 113 normal tissues. (b) Association between PPAR activity and age or clinical stage, with significance determined by the Wilcoxon rank-sum test. (c) Relationship between PPAR activity and overall survival, with significance determined by the Kaplan–Meier analysis and log-rank test. (d) Waterfall plots showing the top 20 genes with the highest mutation frequencies in the high and low PPAR activity groups. (e) Difference in mutation frequency of PCLO between the high and low PPAR activity groups, with significance determined by Fisher's exact test. (f) Pearson's correlations between PPAR activity and TMB, MSI, HRD, stemness indices mDNAsi and mRNAsi, or the expression of MKI67, PCNA, and MCM2. ⁣^∗^*p* < 0.05, ⁣^∗∗^*p* < 0.01, ns: not significant.

The transcriptome data of patients with TNBC from TCGA revealed that the expression of most genes belonging to the PPAR signaling pathway was lower in primary tumors than in normal tissues (Figure 1(a), left). Consistently, the GSVA enrichment score indicated that the PPAR signaling pathway had lower activity in primary tumors compared to normal tissues (Figure 1(a), right). It is worth noting that some TNBC tissues exhibited relatively high PPAR activity (Figure 1(a), right). Indicated by the heterogeneity of PPAR activity in TNBC population, we wondered if alterations in PPAR signaling pathway activity have any clinical significance. In our analysis, we observed that the older group exhibited higher PPAR activity (Figure 1(b)). However, we did not find a significant correlation between PPAR activity and disease stage (stage, T, N, and M) or prognosis (Figures 1(b) and 1(c)). Visualization of the mutational landscape highlighted a significantly higher rate of piccolo presynaptic cytomatrix protein (PCLO) mutations in TNBC patients with higher PPAR activity (Figures 1(d) and 1(e)). Furthermore, we found no link between PPAR activity and multiple oncogenic characteristics, including TMB, MSI, HRD, and stemness (Figure 1(f), left). As for tumor proliferation activity, PPAR activity showed no significant correlation with the expression of proliferation markers (MKI67, PCNA, and MCM2) either (Figure 1(f), right).

### 3.2. Connections Between PPAR Activity and Oncogene Expression or KEGG Pathway Activity in TNBC

**Figure 2:**
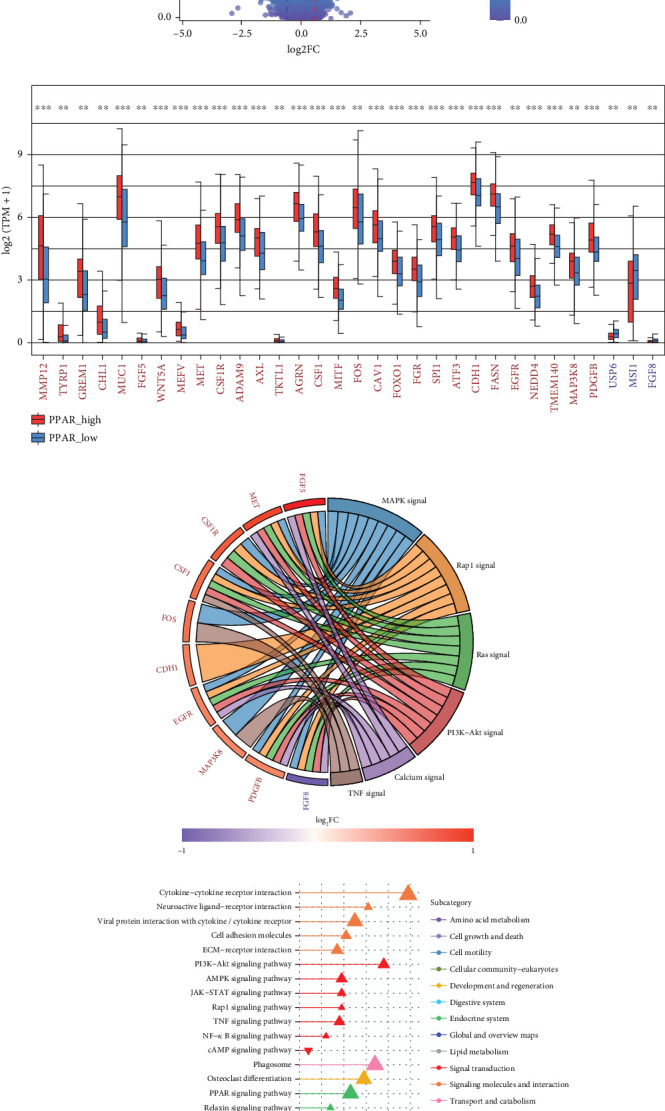
Connections between PPAR activity and oncogene expression or KEGG pathway activity in TCGA-TNBC cohort. (a) Volcano plot displaying the transcriptomic changes between the high and low PPAR activity groups with the differentially expressed oncogenes highlighted. (b) Difference in expression of oncogenes between the high and low PPAR activity groups, with significance determined by the Wilcoxon rank-sum test. ⁣^∗∗^*p* < 0.01, ⁣^∗∗∗^*p* < 0.001. ORA-based KEGG pathway enrichment analysis for (c) differential oncogenes and (d) up- or downregulated genes between high and low PPAR activity groups.

A total of 1056 differentially expressed (799 up- and 257 downregulated) genes, including 29 up- and 3 downregulated oncogenes, were identified in the high compared to low PPAR activity group in the TCGA-TNBC cohort (Figures 2(a) and 2(b)). KEGG pathway enrichment analysis showed that these differential oncogenes were involved in six signal transduction–related pathways, including MAPK, Rap1, Ras, PI3K-Akt, calcium, and TNF signaling pathways (Figure 2(c)). As for the 799 upregulated genes, they were mapped to multiple pathways, such as cytokine–cytokine receptor interaction, PI3K-Akt signaling pathway, phagosome, osteoclast differentiation, PPAR signaling pathway, focal adhesion, regulation of actin cytoskeleton, protein digestion and absorption, tyrosine metabolism, fatty acid metabolism, ferroptosis, and fatty acid biosynthesis (Figure 2(d)). In contrast, the 257 downregulated genes were found to be enriched only for the cAMP signaling pathway (Figure 2(d)). Incidentally, it is worth noting that the upregulation of the PPAR signaling pathway as expected proved the accuracy of GSVA algorithm in assessing PPAR activity.

### 3.3. Relationships Between PPAR Activity and Tumor Immune Microenvironment in TNBC

**Figure 3:**
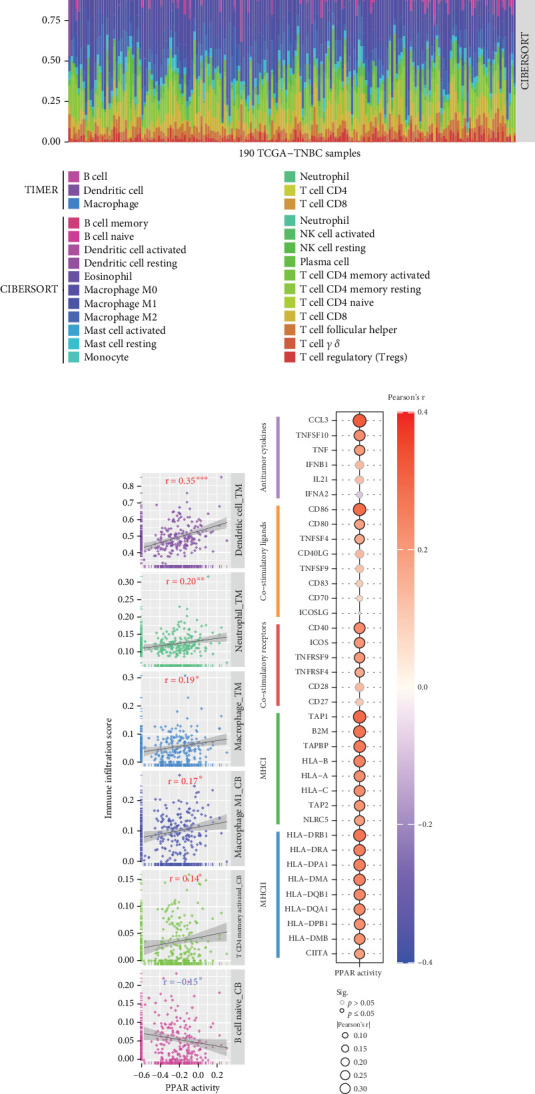
Relationships between PPAR activity and tumor immune microenvironment in TCGA-TNBC cohort. (a) Pearson's correlations between PPAR activity and StromalScore, ImmuneScore, ESTIMATEScore, or tumor purity. (b) Immune cell composition assessed by TIMER and CIBERSORT algorithms. (c) Pearson's correlations between PPAR activity and infiltration scores of dendritic cells, neutrophils, macrophages, M1 macrophages, activated CD4+ memory T cells, or naive B cells. (d) Pearson's correlations between PPAR activity and immune-related gene expression. (e) GSEA plots of nine immune-related pathways significantly enriched in the high compared to low PPAR activity group. (f) Pearson's correlations between PPAR activity and scores of TCR Shannon or CYT. ⁣^∗^*p* < 0.05, ⁣^∗∗^*p* < 0.01, ⁣^∗∗∗^*p* < 0.001.

In the TCGA-TNBC TME, PPAR activity was positively correlated with the infiltration scores of stromal cells (StromalScore), overall immune cells (ImmuneScore), and their combination (ESTIMATEScore), while negatively correlated with tumor purity (Figure 3(a)). Using TIMER and CIBERSORT algorithms, we further estimated the cellular composition of immune infiltrates (Figure 3(b)). Notably, PPAR activity was positively correlated with the infiltration levels of dendritic cells, neutrophils, macrophages, M1 macrophages, and activated CD4+ memory T cells but negatively correlated with that of naive B cell (Figure 3(c)). Dendritic cells and M1 macrophages are well known to contribute to antitumor immunity to a large extent [43]. In addition, PPAR activity demonstrated a significant positive correlation with the expression of numerous immune-related genes (Figure 3(d)). Consistently, nine immune-related signaling pathways were strongly enriched in the group with higher PPAR activity (Figure 3(e)). Correspondingly, increased PPAR activity was accompanied by significant increases in TCR Shannon diversity and immune CYT (Figure 3(f)). These results highlighted the likelihood of PPAR signaling in eliciting anti-TNBC immunity.

### 3.4. Associations Between PPAR Activity and TME-Resident Microorganisms in TNBC

**Figure 4:**
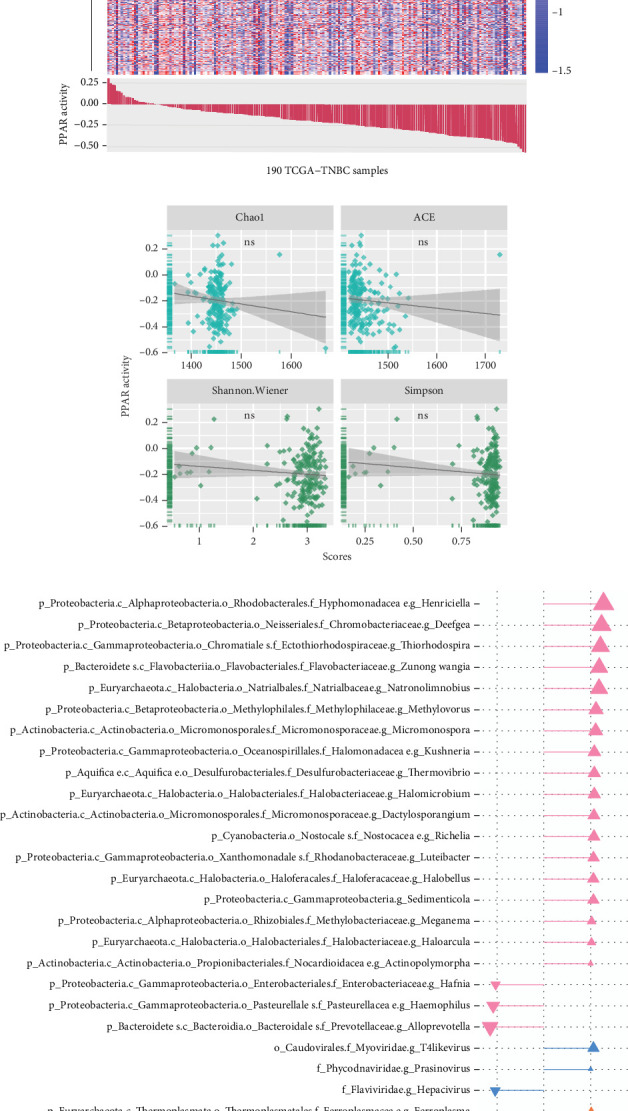
Associations between PPAR activity and TME-resident microorganisms in TCGA-TNBC cohort. (a) Distribution pattern of 1406 microorganisms in 190 TNBC samples. (b) Pearson's correlations between PPAR activity and scores of Chao1, ACE, Shannon, or Simpson. ns: not significant. (c) Pearson's correlations between PPAR activity and specific microorganism content.

The TME is also a favorable niche for microbial survival and growth [20]. Considering that both PPAR signaling pathway and intratumoral microbiota have a significant impact on cancer development through the regulation of metabolism, further investigation is required to explore and understand the potential connections between them [21]. Really, in the TCGA-TNBC TME, there were a vast diversity of microorganisms (Figure 4(a)). Regrettably, no significant relationship was found between PPAR activity and microbial alpha diversity, including species richness (Chao1 and ACE) and diversity (Shannon and Simpson) (Figure 4(b)). Interestingly, however, PPAR activity showed a positive correlation with 21 microorganisms, comprising 18 bacteria, 2 viruses, and 1 archaea, while it was negatively correlated with 5 microorganisms, including 3 bacteria, 1 virus, and 1 archaea (Figure 4(c)). The genera *Henriciella*, *T4-like virus*, and *Ferroplasma* were identified as the most positively correlated bacterium, virus, and archaea, respectively. In contrast, the genera *Alloprevotella*, *Hepacivirus*, and *Pyrolobus* emerged as the most negatively correlated bacterium, virus, and archaea, respectively. These findings suggested a potential relationship between PPAR activity and colonization of specific microorganisms in the TNBC TME.

### 3.5. Drug Sensitivity Correlation of PPAR Activity and Prediction of Drugs Modulating It

**Figure 5:**
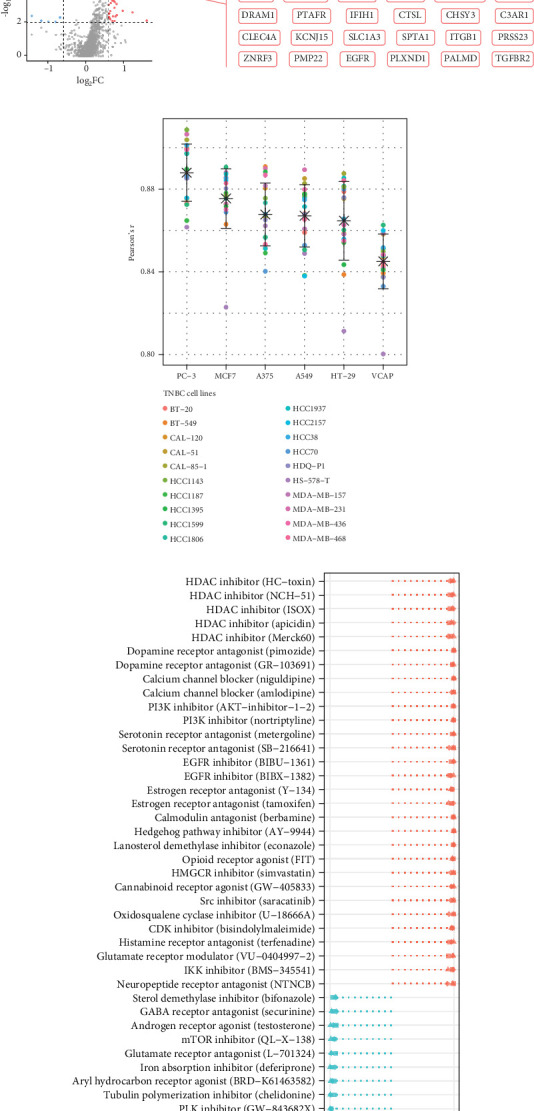
Drug sensitivity correlation of PPAR activity and prediction of drugs modulating it. (a) Pearson's correlations between PPAR activity and IC50 values of specific drugs in GDSC-TNBC cells. ⁣^∗^*p* < 0.05, ⁣^∗∗^*p* < 0.01. (b) Volcano plot displaying the transcriptomic changes of 938 chemoresistance-related genes between the high and low PPAR activity groups in TCGA-TNBC cohort. (c) Pearson's correlations among the gene expression profiles of 6 CMap-derived non-TNBC and 20 GDSC-derived TNBC cell lines. (d) Identified potential compounds that may regulate the PPAR signaling pathway in TNBC by CMap analysis.

We next utilized the GDSC dataset to explore the relationship between PPAR activity and sensitivity to anticancer drugs in TNBC cell lines. Notably, PPAR activity exhibited a positive correlation with the IC50 values of 10 drugs, including several chemotherapeutic drugs (teniposide, vinblastine, camptothecin, and epirubicin), as well as dactolisib, Wee1 inhibitor, AZD1332, BI-2536, MK-1775, and luminespib (Figure 5(a)). Conversely, PPAR activity showed a negative correlation with the IC50 values of 14 drugs, such as oxaliplatin, venetoclax, EPZ5676, gefitinib, XAV939, picolinic acid, AZD3759, ibrutinib, osimertinib, ruxolitinib, erlotinib, savolitinib, MN-64, and LY2109761 (Figure 5(a)). We also noted that higher PPAR activity seemed to come with upregulation of 36 chemoresistance-related genes in the TCGA-TNBC cohort (Figure 5(b)).

Additionally, we focused on drug perturbations. We conducted a CMap analysis to identify potential compounds that can activate or inhibit the PPAR signaling pathway. Due to the limited availability of TNBC cell line data in the CMap database, we selected three available non-TNBC cell lines (PC-3, MCF7, and A375) that closely resemble TNBC cell lines in their gene expression profiles for further analysis (Figure 5(c)). According to the results of prediction and analysis, 30 candidate compounds may act as activators of the PPAR signaling pathway, while nine compounds could serve as its inhibitors in the context of TNBC (Figure 5(d)). Notably, up to five HDAC inhibitors were identified as drug candidates for upregulating PPAR activity in TNBC.

### 3.6. Upregulation of PPAR Signaling Pathway by HDAC Inhibitor Chidamide in Human TNBC MDA-MB-231 Cells

**Figure 6:**
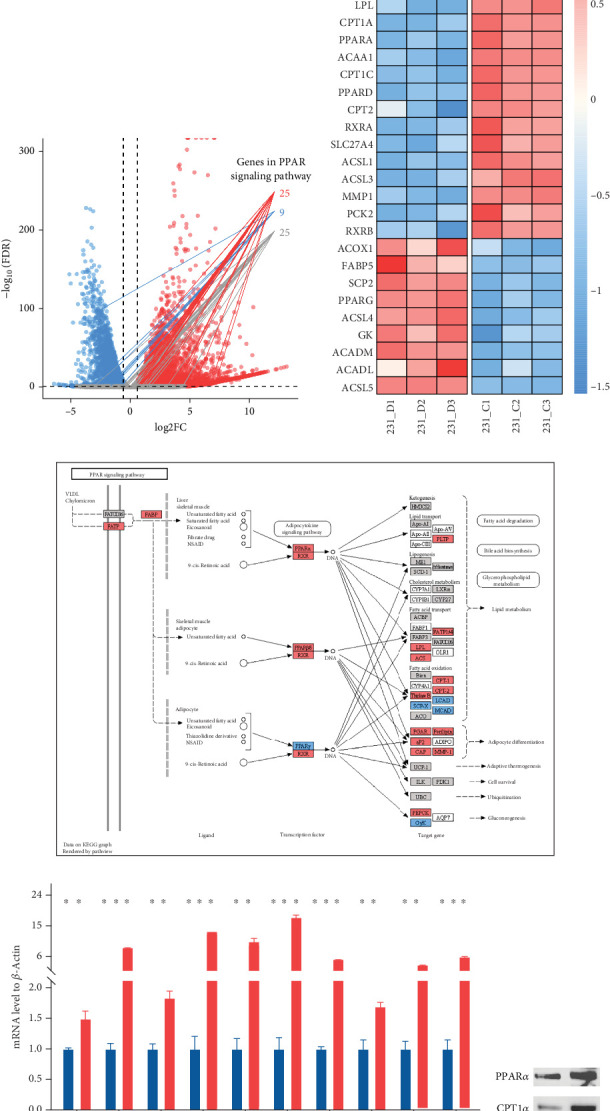
Upregulation of the PPAR signaling pathway by HDAC inhibitor chidamide in human TNBC MDA-MB-231 cells. (a) Volcano plot displaying the transcriptomic changes in MDA-MB-231 cells following chidamide treatment with the genes involved in the PPAR signaling pathway highlighted. Dots representing 25 upregulated, 9 downregulated, and 25 nondifferential genes were colored in red, blue, and gray, respectively. (b) Heat map displaying the expression patterns of 25 upregulated and 9 downregulated genes involved in the PPAR signaling pathway from (a). (c) Pathview mapping result of genes involved in the PPAR signaling pathway from (a), with rectangles indicating upregulated, downregulated, and nondifferential genes filled in red, blue, and gray, respectively. (d) qRT-PCR analysis for 10 representative genes, with significance determined by Student's *t*-test. ⁣^∗∗^*p* < 0.01 and ⁣^∗∗∗^*p* < 0.001. (e) Western blot analysis for PPAR*α* and CPT1*α*.

The CMap analysis suggested that the PPAR signaling pathway can be regulated by certain drugs, especially HDAC inhibitors, in the context of TNBC. To verify this prediction, we performed RNA-seq in human TNBC MDA-MB-231 cells either treated or untreated with chidamide, one of only two currently approved HDAC inhibitors for solid tumor treatment worldwide. As anticipated, our differential expression analysis revealed that treatment with chidamide resulted in upregulation of 25 and downregulation of 9 genes involved in the PPAR signaling pathway (Figures 6(a) and 6(b)). Pathview mapping of these detected genes from the PPAR signaling pathway further illustrated the biological functions that they may perform (Figure 6(c)). Specifically, the upregulated genes are indicated to encode the core regulators (PPAR*α*: PPARA, PPAR*β*/*δ*: PPARD; RXR: RXRA and RXRB) of the pathway, along with proteins (FATP: SLC27A1 and SLC27A2; FABP: FABP4 and FABP6) responsible for transporting fatty acids to activate the aforementioned transcriptional regulators. Furthermore, upregulated genes also involve downstream proteins associated with lipid metabolism (PLTP: PLTP; LPL: LPL; ACS: ACSL1, ACSL3, and ACSBG1; CPT-1: CPT1A and CPT1C; CPT-2: CPT2; Thiolase B: ACAA1), adipocyte differentiation (PGAR: ANGPTL4; Perilipin: PLIN4 and PLIN5; aP2: FABP4; CAP: SORBS1; MMP-1: MMP1), and gluconeogenesis (PEPCK: PCK2). To strengthen the reliability of the above results, we verified the gene expression changes of 10 representative genes (PPARA, CPT1A, etc.) by qRT-PCR (Figure 6(d)). In addition, Western blot analysis of PPAR*α* and CPT1*α* further confirmed the protein expression changes (Figure 6(e)). Collectively, these results underscore the regulatory role of chidamide in modulating key elements of the PPAR signaling pathway, which is essential for lipid metabolism, adipocyte differentiation, and gluconeogenesis.

## 4. Discussion

The PPAR signaling pathway is involved in various physiological processes, including glucose metabolism, lipid metabolism, cell differentiation, proliferation, inflammation, and vascular biology. Additionally, it plays a role in metabolic reprogramming that can promote cancer progression [44, 45]. The development of effective treatments for TNBC is significantly challenged by its high heterogeneity. Currently, little is understood about how the PPAR signaling pathway contributes to the phenotypic and molecular heterogeneity observed in TNBC.

Retrospective analysis could help uncover pathogenic abnormalities and optimize therapeutic strategies [46–48]. In this study, we retrospectively examined the clinical relevance of the PPAR signaling pathway within TNBC patients. Regrettably, our findings indicated that PPAR pathway activity did not correlate with clinical stage, prognosis, TMB, MSI, HRD, stemness, or proliferation status. Nevertheless, interestingly, its increase was associated with the older age of TNBC patients. This finding is surprising, as organismal aging is typically characterized by reduced PPAR signal in various tissues, including the kidney, liver, and heart [49, 50]. Furthermore, we observed that high PPAR activity correlated with mutations in the PCLO gene, which encodes a protein involved in synaptic function and has been linked to oncogenic signaling in cancer like EGFR signaling in esophageal squamous cell carcinoma [51]. The relationship between PPAR signal and PCLO in TNBC warrants further investigation.

Aberrant expression of oncogenes is a well-known reason for tumor initiation and progression [52]. Notably, elevated PPAR activity was also associated with the upregulation of oncogenes involved in several signaling pathways, including MAPK, Rap1, Ras, PI3K-Akt, calcium, and TNF. This suggests that PPAR activation may play a role in oncogenic signal transduction in TNBC. Moreover, PPAR signal positively correlated with pathways related to fatty acid metabolism, fatty acid biosynthesis, AMPK signaling, ferroptosis, and so forth, while negatively correlated with the cAMP signaling pathway. Indeed, in breast cancer, the involvement of the PPAR signaling pathway in regulating fatty acid synthesis, oxidation, uptake, and activation is well documented [53]. In non–small cell lung cancer, pharmacological activation of PPAR signal has been shown to activate the AMPK signaling pathway [54]. Another recent study demonstrated that PPAR signal can promote ferroptosis by upregulating the expression of ACSL1/4 in pancreatic ductal adenocarcinoma [55]. However, more evidence is needed to establish a causal link with the other identified pathways. As for the other identified pathways during our study, more studies are needed to provide clearer evidence in this regard.

The TME is rich in both immune and stromal cells [56]. Our analysis revealed that higher PPAR activity was associated with increased levels of infiltrating immune and stromal cells in the TNBC TME thereby resulting in lower tumor purity. Specifically, PPAR activity displayed a positive correlation with the infiltration of classical antitumor immune cells, including dendritic cells and M1 macrophages, as well as with the expression of antitumor immune-related molecules, including cytokines, costimulatory factors, and MHCs. Moreover, the increase in immune-related pathway enrichment, TCR diversity, and CYT activity in the high PPAR activity group implied a potential role for PPAR signal in promoting anti-TNBC immunity. Supporting our findings, a previous study indicated that activating PPAR*γ* with its agonist rosiglitazone increased M1 macrophage numbers in the small intestine of mice [57]. Additionally, PPAR*α* agonist fenofibrate and PPAR*γ* agonist pioglitazone have been shown to enhance the antitumor activity of cytotoxic T lymphocytes and invariant natural killer T cells in melanoma, respectively [58, 59]. Pharmacological activation of PPARs can also effectively improve the efficacy of cancer vaccine and adoptive cell therapy [60–62]. What is more, a clinical trial (UMIN000017854) has even suggested that combining the pan-PPAR agonist bezafibrate with the PD-1 immune checkpoint inhibitor nivolumab could improve treatment outcomes in non–small cell lung cancer [63]. Thus, future researches can focus on leveraging PPAR signal to boost the antitumor immune responses in TNBC.

As cancer research progresses, it has become evident that tumor tissues previously thought to be sterile contain various microbiota [64], which can affect cancer development or the anticancer efficacy through various mechanisms. Alpha diversity serves as an important measure of microbial balance and stability [65]. Regrettably, we found no significant association between PPAR activity and microbial alpha diversity in TNBC tissues. Nevertheless, we noted the positive correlations between PPAR activity and the abundance of certain microorganisms such as those belonging to the genera *Micromonospora*, *Haloarcula*, and *Luteibacter* and the negative correlations with others like genera *Haemophilus*, *Alloprevotella*, and *Hepacivirus*. Notably, thiocoraline, derived from genus *Micromonospora*, has been found to inhibit breast cancer cell growth [66, 67]. Similarly, carotenoid pigment from genus *Haloarcula* can induce apoptosis of breast cancer cells [68]. Genus *Luteibacter* was found to be associated with disease progression in lung adenocarcinoma [69]. In contrast, the presence of salivary genus *Haemophilus* has been linked to an increased risk of breast cancer [70], and infection of hepatitis C virus, which belongs to the genus *Hepacivirus*, is associated with the development and poor chemotherapy outcomes in breast cancer [71–73]. Genus *Alloprevotella* has been regarded as a cancer-related bacteria in various types of cancer such as oral cancer [74, 75], glottic laryngeal squamous cell carcinoma [76], thyroid cancer [77], pancreatic cancer [78], colon cancer [79], and cervical cancer [80]. Overall, increased PPAR activity appeared to support the formation of an antitumor microbial microenvironment in TNBC patients.

Another significant challenge in cancer treatment is resistance to cytotoxic drugs [81]. Chemotherapy remains a primary treatment modality for TNBC [82]. We found that increased PPAR activity was associated with upregulation of certain chemoresistance-related genes and resistance in TNBC cells against commonly used chemotherapeutic drugs, including camptothecin, vinblastine, epirubicin, and teniposide. Our recent review has also highlighted the activation of the PPAR signaling pathway as a factor contributing to resistance against various anticancer treatments, including chemotherapy [83]. In addition, we identified several potential drug candidates capable of modulating the PPAR signal. In particular, our experiments confirmed that treatment with HDAC inhibitor chidamide can activate PPAR signal in TNBC cells. Notably, a clinical trial observed that the addition of chidamide did not improve the effectiveness of chemotherapeutic drug cisplatin in TNBC patients [84]. Our current findings may help partly explain such disappointing results. As for tumor immunity, chidamide was shown to significantly enhance tumor immunogenicity and boost antitumor immunity in TNBC [85]. Given our findings that increased PPAR activity was associated with the formation of an antitumor immune microenvironment in TNBC patients, this warrants further investigation to determine whether the immunostimulatory effects of chidamide are in part attributed to its activation of the PPAR signaling pathway. Exploring this connection could provide valuable insights for improving therapeutic strategies in TNBC.

These findings suggest promising avenues for future research in understanding and targeting PPAR signal in TNBC. However, it is crucial to acknowledge the limitations in our study. For instance, the causal relationships between PPAR activity and the various biological aspects that collectively influence cancer development and treatment response in TNBC, such as the expression of identified oncogenic genes and signaling pathways, antitumor immune-related molecules and pathways, infiltration of antitumor cells, abundance of specific microorganisms, and expression of certain chemoresistance-related genes, need further clarification. Additionally, due to the retrospective nature of the study and potential population heterogeneity, further studies are needed to validate the robustness of our results in different TNBC populations.

## 5. Conclusion

This comprehensive analysis depicts the clinical significance and potential drug modulation of the PPAR signaling pathway in TNBC. Increased PPAR activity was associated with older age, a higher frequency of PCLO mutations, and oncogenic signaling pathways such as MAPK, Ras, and PI3K-Akt, as well as biological processes like fatty acid metabolism, AMPK signaling, and ferroptosis. Besides, elevated PPAR activity appeared to promote the formation of an antitumor immune and microbial microenvironment. Furthermore, high PPAR activity was associated with resistance to common chemotherapeutic drugs in TNBC cells. Our analysis also identified several promising compounds, including HDAC inhibitors, which could modulate PPAR signal in TNBC. In conclusion, PPAR signal likely has pleiotropic biological effects in TNBC. While these findings require validation through larger studies, they indicate important avenues for future research in understanding and targeting PPAR signal in TNBC.

## Data Availability

The public datasets analyzed in the current study are available in online repositories as described in Materials and Methods. Besides, our RNA-seq raw data have been deposited and can be accessed at https://www.ncbi.nlm.nih.gov/bioproject/PRJNA1090785.
